# Die sexuelle Revolution und der Aufstieg der Urologen in der Bundesrepublik

**DOI:** 10.1007/s00120-025-02549-x

**Published:** 2025-03-12

**Authors:** Florian G. Mildenberger

**Affiliations:** Institut für Geschichte, Theorie und Ethik der Medizin, Heinrich-Heine Universität Düsseldorf, Düsseldorf, Deutschland

**Keywords:** Allgemeinmediziner, Sexualaufklärung, Sexualwissenschaft, Urologie, Sexuelle Revolution, Paul Lüth, Albert Moll, General practitioners, Sexual education, Sexology, Urology, History of urology, Sexual revolution, Paul Lüth, Albert Moll

## Abstract

Die sexuelle Revolution der 1960er-Jahre wird gemeinhin nicht mit der Urologie assoziiert. Jedoch sorgte die allgemeine Verfügbarkeit sexueller Dienstleistungen und die Inkompetenz, fehlende Vergütungsmöglichkeiten für die Fachgruppe oder fachliches Desinteresse der Allgemeinmediziner, damit und mit den veränderten gesellschaftlichen Rahmenbedingungen umzugehen, dazu, dass die Fachärzte wieder zu den „Herren des Unterleibs“ wurden. Dadurch wurde ein 1933 abgerissener Diskurs neu geknüpft. Heute haben die Urologen längst die Hausärzte als zentrale Ansprechpartner, Diagnostiker und Therapeuten in Fragen der Männergesundheit abgelöst.

## Abstract (en)

The sexual revolution of the 1960s is not generally associated with urology. However, the general availability of sexual services combined with the lack of expertise of general practitioners to deal with this and the changed social conditions led to urologists once again becoming the “masters of the genital area”. This reopened a discourse that had been interrupted in 1933. Today, urologists have long since replaced GPs as the primary point of contact, diagnosticians and therapists for men’s health issues.

## Rückblick und Einleitung

**Abb. 1:**
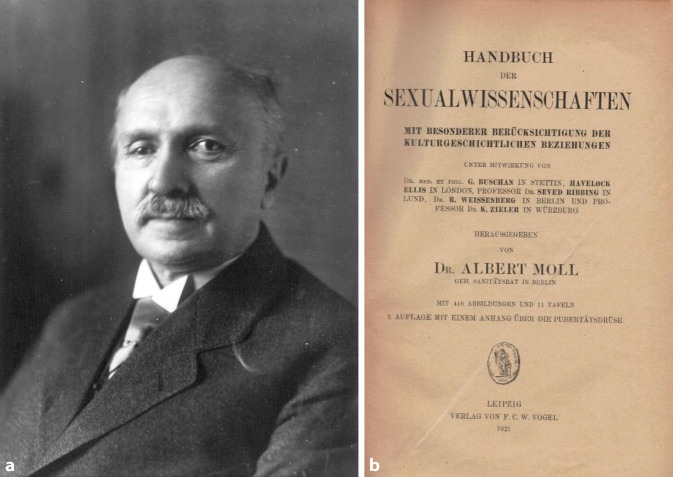
**a** Albert Moll (1862–1939) ca. 1927, Ullstein Bild, **b** Frontispiz Handbuch der Sexualwissenschaften 2. Auflage 1921 bei FCW Vogel in Leipzig. (Repro Moll-Keyn, Sammlung Moll, mit freundl. Genehmigung)

**Abb. 2:**
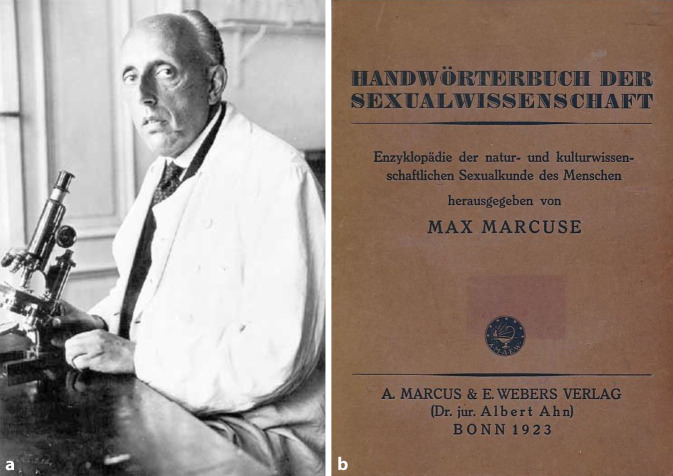
**a** Max Marcuse, **b** Frontispiz Handwörterbuch der Sexualwissenschaft Enzyklopädie der natur- und kulturwissenschaftlichen Sexualkunde des Menschen. Ausgabe 1923. Hier war auch der Berliner Urologe Carl Posner (1854–1928), Berlin, Beiträger. (Repro Moll-Keyn, Sammlung Moll, mit freundl. Genehmigung)

**Abb. 3:**
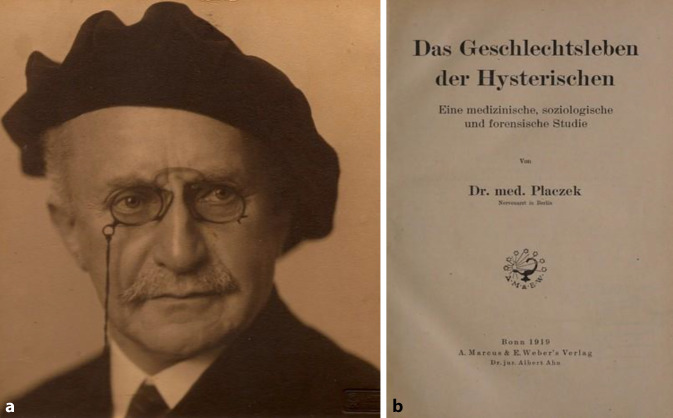
**a** Siegfried Placzek (1866–1946) wikicommons, **b** Frontispiz Geschlechtsleben der Hysterischen. Ausgabe 1919. Placzek gehörte zu den wenigen Autoren die bereits vor dem Jahre 1900 eine ausführliche Beschreibung des Amerikanischen Krankenhauswesens lieferte, zu einer Zeit, als die Besucherströme auf Europa gerichtet waren. (Repro Moll-Keyn, Sammlung Moll, mit freundl. Genehmigung)

In den 1920er-Jahren erlebte die noch junge Sexualwissenschaft im Deutschen Reich einen ungeahnten Höhenflug. In Berlin fungierte das von Magnus Hirschfeld (1868–1935) finanzierte und organisierte „Institut für Sexualwissenschaft“ als Denkfabrik [1, 2]. Daneben wirkten in eigener Praxis, aber ebenfalls mit umfänglicher Forschung niedergelassene Spezialisten wie Albert Moll (1862–1939) (Abb. 1), Max Marcuse (1877–1963) (Abb. 2) oder Siegfried Placzek (1866–1946; [3–5]) (Abb. 3). Trotz ihrer persönlichen Differenzen sorgten sie dafür, dass Sexualaufklärung, sexuelle Gesundheitsberatung, Hilfe bei ungewollter Schwangerschaft oder im Falle der Infektion mit Geschlechtskrankheiten nicht mehr, wie bisher so häufig, durch Allgemeinmediziner („Hausärzte“) erfolgte, sondern dass die Patienten ihrerseits den Weg in die Praxis von Fachärzten suchten.

Diese Entwicklung ging einher mit einer zunehmenden Spezialisierung der Ärzte insgesamt. Es war mit Max Hodann (1894–1946) ein Mitstreiter Hirschfelds, der die Sexualaufklärung für Erwachsene professionalisierte und so den Fachärzten für Frauenheilkunde und Urologie (ungewollt) einen erheblichen Zufluss an Patienten bescherte [6]. Parallel jedoch entfaltete sich insbesondere in der zweiten Hälfte der 1920er-Jahre eine Gegenbewegung. Denn die medizinischen Leitwissenschaften in Deutschland, die vor 1918 den baldigen Sieg im Kampf gegen Krebs, Infektionskrankheiten, Syphilis und Tuberkulose versprochen hatten, konnten diese Ankündigungen nicht einhalten. Zugleich sorgte die zunehmende Zahl an jungen Ärzten, allgemeine Ressourcenverknappung und die Folgen der Inflation für Verarmungsängste innerhalb der Ärzteschaft. In Fachöffentlichkeit und im populären Diskurs wurde immer häufiger von einer „Krisis der Medizin“ gesprochen [7, 8]. Es waren gerade die Arbeiten des Danziger Gynäkologen Ernst Liek (1878–1935) und seines Wiener Kollegen Bernhard Aschner (1883–1960), die der Debatte eine entscheidende Wendung verliehen [9, 10]. Beide Ärzte kritisierten das zunehmende „Spezialistentum“ innerhalb der Medizin und forderten eine Rückkehr zu einer ganzheitlichen Heilkunde, die weniger auf Apparate oder teure Arzneien setzte sondern eher das vertrauliche Arzt-Patienten-Gespräch und damit die psychosomatische Ebene in den Mittelpunkt rückte. Dies beförderte den Hausarzt zurück ins Zentrum der Familienmedizin. Der Industrielle Robert Bosch (1861–1942) förderte einerseits die Homöopathie, andererseits aber die ganzheitlich orientierte Allgemeinmedizin. Zu seinen Projekten gehörte u. a. der „Hippokrates-Verlag“, der 1925 die Zeitschrift *Der Landarzt* lancierte, die (neben dem Journal *Hippokrates*) zum Leitmedium der Krisis-Ärzte wurde. Nach 1933 erfolgte eine Ideologisierung der Debatte. So wurden insbesondere die Akteure der Sexualwissenschaft aufgrund ihrer jüdischen Herkunft und/oder sozialistischen Einstellung aus dem Diskurs verbannt und aus Deutschland vertrieben [11]. Der sich daraus entwickelnde Mangel an Fachärzten suchte das Regime durch eine Synthese verschiedener Heilkulturen zu kompensieren, deren Erarbeitung einer 1935 aus der Taufe gehobenen „Reichsarbeitsgemeinschaft für eine Neue Deutsche Heilkunde“ oblag [12]. Dies trug neben der Forderung zur Ressourcenschonung zu einer Stärkung der zu ganzheitlicher Diagnose und Therapie angehaltenen Allgemeinmediziner bei. Deren Arbeit wurde nach 1945 durch die Einführung von Cortison und Antibiotika bedeutend erleichtert. Diese neuen Medikamente trugen dazu bei, dass Patienten, die bislang allein von Fachärzten behandelt wurden, nun den Hausärzten erhalten blieben. Diese positionierten sich selbst als Hüter von Familiengesundheit und der bestehenden sittlichen Ordnung.

## Krisenerscheinungen und Kritik

Diese Selbstpositionierung war nicht auf Deutschland beschränkt, auch in den USA sahen sich die Allgemeinmediziner als in allen Belangen der Heilkunde bewanderte Hüter der Volksgesundheit. Doch die Rezeption des Kinsey-Reports trug in den USA dazu bei, dass Sozialmediziner begannen, das Selbstbild der niedergelassenen Ärzte kritisch zu hinterfragen und zu überprüfen, ob sie wirklich über Fachkenntnis in den Bereichen Familien- und Sexualberatung verfügten [13–15]. Denn Studien hatten ergeben, dass es gerade sexuelle Probleme waren, die scheinbar gesunde Menschen in ärztliche Praxen trieben [15]. Es offenbarte sich, dass die vorgebliche Kompetenz der Allgemeinärzte in keiner Weise bestand und ihr Selbstbild als Helfer der Familien zwar propagandistisch wirksam vertreten wurde, aber nicht durch Fakten begründet war. In der Bundesrepublik Deutschland konnte sich jeder Jungmediziner nach bestandenen Staatsexamen als „Arzt für Allgemeinmedizin“ oder gar „Arzt für die gesamte Heilkunde“ niederlassen, ohne irgendwelche Fortbildungen besuchen zu müssen [16]. Erst auf dem 59. Deutschen Ärztetag 1956 in München wurde das Thema erstmals diskutiert, doch Pflichtfortbildungen erfolgten erst schrittweise nach 1961. Deren Steuerung übernahm der als besonders konservativ geltende Bayerische Ärztekammerpräsident Hans-Joachim Sewering (1916–2010; [17]). Ab 1964 fanden die „Heidelberger Gespräche“ zwischen Klinikern und Vertretern der Hausärzte statt, um letztere zu professionalisieren. Eine zentrale Rolle bei der Stärkung des eigenen Selbstbildes kam dem österreichischen Landarzt Robert Nikolaus Braun (1914–2007) zu [16]. Er veröffentlichte eine Reihe von Schriften, die den niedergelassenen Ärzten nicht nur ihre diagnostische und therapeutische Arbeit erleichtern sollten, sondern auch ihr Selbstbild als Hüter der Familiengesundheit stärken würden. Doch Sexualität, Sexualaufklärung oder auch nur psychosomatische Ansätze spielten darin abseits von Geschlechtskrankheiten keine Rolle [18, 19]. Aber die Zeiten änderten sich.

## Die sexuelle Revolution und die ratlosen Hausärzte

**Abb. 4:**
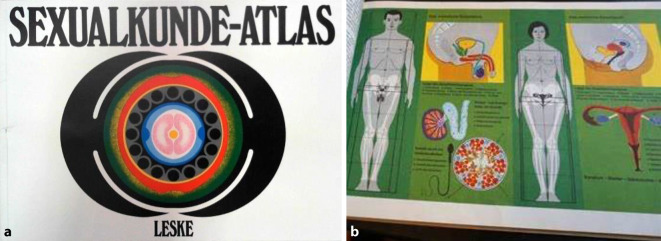
**a** Sexualkundeatlas 1969, Leske Verlag. **b** Die Abbildungen können heute mehr zu einer Geschichte der Informationsgrafik als zu einer Geschichte des Sexualkundeunterrichtes dienen. (Repro Moll-Keyn, Sammlung Moll, mit freundl. Genehmigung)

In 1961 war die Antibabypille auf den Markt gekommen und wurde sukzessive verschrieben [20]. Dazu trug die katholische Kirche ungewollt bei, weil einer ihrer führenden Moraltheologen, Bernhard Häring (1912–1998) ausgeführt hatte, dass dieses Medikament mit der päpstlichen Enzyklika „Casti Connubii“ vereinbar sei, woraufhin hunderte von Priestern ihren Beichtkindern die Einnahme der „Pille“ empfahlen [21, 22]. Dies sollte sich 1968 nach Erlass der Enzyklika „Humanae Vitae“ als Problem herausstellen. Aus Skandinavien schwappte zunehmend die „Porno-Welle“ ins Bundesgebiet, während Anbieter wie Beate Uhse (1919–2001) oder die Briefkastenfirma „Pharmawerk Schmiden“ im Postversand interessierte Kunden mit Produkten des erotischen Marktes versorgten [23, 24]. Dies alles kulminierte am Ende des Jahrzehnts in Form der Entkriminalisierung des Sexualstrafrechts, woraufhin eine bislang ungeahnte sexuelle Freiheit in der vormaligen Adenauerrepublik ihren Lauf nahm. Dies beinhaltete auch eine Verschiebung der Haltung zu Autoritäten. „Jugendsexualität“ avancierte zu einem Thema, auf das die Ärzteschaft wenig vorbereitet war [25]. 1967 wurde die schulische Sexualaufklärung verankert und damit schien den Ärzten eine wichtige Einflussmöglichkeit genommen (Abb. 4).

**Abb. 5:**
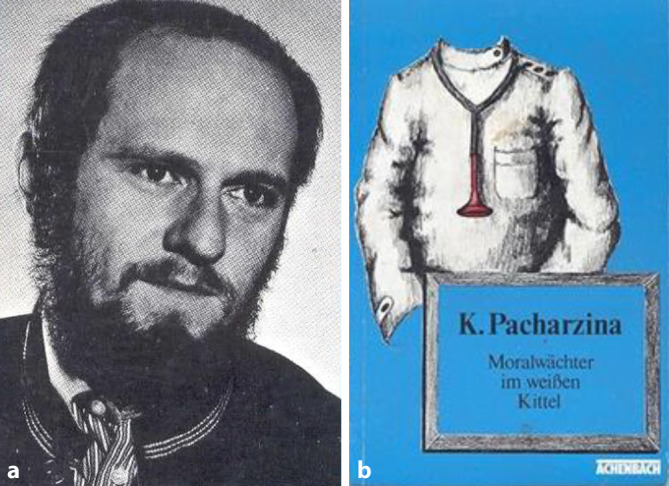
**a** Klaus Pacharzina (geb 1947) Moralwächter im weißen Kittel. Zur Sexualmedizin in der Allgemeinpraxis, **b** Frontispiz. (Repro Moll-Keyn, Sammlung Moll, mit freundl. Genehmigung)

Die Hausärzte sollten nach Ansicht ihrer Standespolitiker dem Patienten gegenüber stets „Überlegenheit“ betonen und niemals „nachgiebig“ sein [26]. Alles andere würde eine „Krise der Medizin“ hervorrufen [27]. Als schwierig wurden die „Namensgebung seelischer Inhalte“ benannt [28]. Noch immer durchwehte die Hausarztpraxis der Geist der Eugenik, die im Zentrum jeder Form von Sexualberatung stehen sollte [29]. Der Veteran der Sexualreformbewegung der 1920er-Jahre und Medizinhistoriker Werner Leibbrand (1896–1974), München, warnte die Ärzteschaft: „Eros und Sexus wird weder von Philosophen noch von Ärzten allein gemacht. Was sie theoretisch zu sagen haben, mag gelegentlich verstanden oder *missverstanden* werden.“ [30]. Die sexuelle Revolution machte vor den Praxen nicht Halt. Trotz schulischer Sexualbelehrung sahen sich die Allgemeinmediziner mit einer wachsenden Zahl an Mädchen und jungen Frauen konfrontiert, die ihre Beziehungsprobleme in die Praxen brachten [31]. Als Standardwerk für die hausärztliche Psychotherapie fungierte gemeinhin das Buch „Die seelische Krankenbehandlung“ von Johannes H. Schultz (1884–1970), der bei einem Umfang von 450 Seiten dem Sexualleben genau eine Seite widmete [32]. Selbstkritisch notierte der in Lühnde bei Hildesheim ansässige Arzt Horst Eckard Wittneben (1943–2021), man müsse in seinem Fach „Psychologie, Theologie, Tiefenpsychologie, Pädiatrie, Sozialwissenschaft, Soziologie, Politik“ beherrschen und dann noch den „Jugend- und Eheberater“ spielen [33]. Das schien nach Ansicht der Funktionäre der Deutschen Gesellschaft für Allgemeinmedizin kein Problem zu sein, aber dann veröffentlichte Klaus Pacharzina (geb. 1947) (Abb. 5), Mitarbeiter am Institut für Sexualwissenschaft der Universität Frankfurt/M. in der Zeitschrift *Sexualmedizin* 1975 Auszüge aus seiner Dissertation. Hierzu befragte er 100 Ärzte aus dem Raum Hannover bezüglich ihrer sexualwissenschaftlichen und psychologischen Kompetenzen [34]. Diese Studie öffnete der Fachöffentlichkeit die Augen: Die Mehrheit der zumeist älteren Herren erhob ihre Privatmeinung über sexuelles Verhalten zur wissenschaftlichen Wahrheit und weigerte sich, Patienten wahlweise die „Pille“ zu verschreiben oder sich weiter zu bilden [35]. In der 1978 veröffentlichten Buchversion der Dissertation offenbarte sich dann noch die totale Inkompetenz der niedergelassenen Ärzte in Fragen der menschlichen Sexualität [36]. Auch zeigte sich, dass bereits die Kenntnis der Nebenwirkungen der „Pille“ zahlreiche Hausärzte restlos überforderte [37].

## Delegation als Lösungsansatz?

**Abb. 6:**
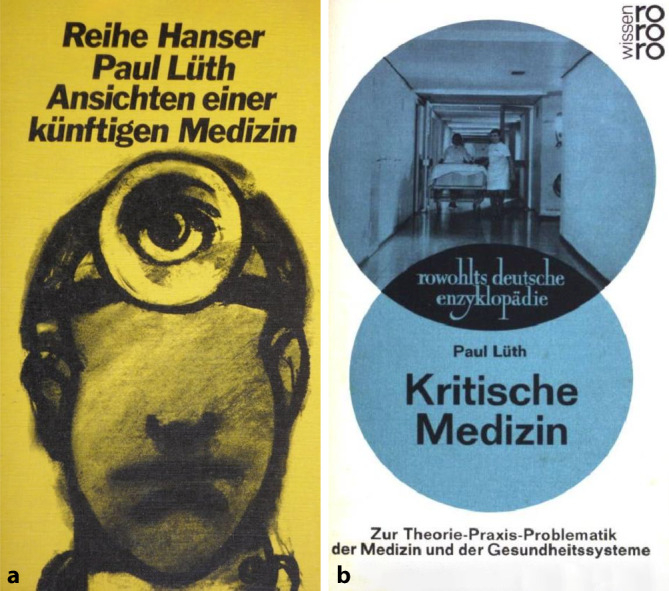
Die Werke von Paul Lüth (1921–1986), Ansichten einer künftigen Medizin, 1971 sowie Kritische Medizin, 1972, prägten eine ganze Ärztegenration. (Repro Moll-Keyn, Sammlung Moll, mit freundl. Genehmigung)

Diese Probleme wurden von Robert N. Braun weiterhin in seinen Büchern ignoriert. Er verstieg sich 1976 sogar zu der Annahme, dass sich die sexuellen Probleme nur in der Stadt und nicht auf dem Lande stellen würden [38]. Auch in späteren Werken klammerte er die menschliche Sexualität als Thema in der Sprechstunde aus [39–41]. An seine Stelle rückte als Ideengeber für eine moderne Allgemeinpraxis der Medizinsoziologe Paul Lüth (1921–1986; [42]) (Abb. 6). Er warnte die Hausärzte, dass sie in Zukunft ihre alleinige Deutungshoheit einbüßen würden, da sie die bis 1967 von den Ärztekammern abgelehnten Gemeinschaftspraxen alsbald beziehen würden [43]. Lüth betonte ausdrücklich, dass der niedergelassene Arzt sich auf die Patienten einlassen müsse und sich in ihre Denkwelt einfügen solle [44].

Ohnehin schockierte die „Konfrontation mit der sozialen Wirklichkeit“ viele junge Landärzte [45]. Aus seiner eigenen Erfahrung wusste Lüth, dass gerade jugendliche Patienten im Fall einer Syphilisbehandlung diese häufig abbrachen, weil sie nicht genügend unterrichtet waren [46]. Folgerichtig ermahnte Lüth seine Leser, „urologische Störungen“ und die Dermatologie als Fachgebiet nicht länger zu vernachlässigen [47]. Ärztliche Autorität, so Lüth, sei kein Selbstläufer, sondern müsse jeden Tag neu errungen werden [48]. Als Ausweg empfahl er eine enge Zusammenarbeit mit den gynäkologischen Fachpraxen, da er davon ausging, dass hier der beste Ort für Sexualberatung sei [46, 49]. Dies führte dazu, dass sich Gynäkologen ab der zweiten Hälfte der 1970er-Jahre zunehmend selbst zu den Hausärzten der Zukunft kürten [47, 50]. In der Praxis jedoch wurden Patienten mit sexuellen Fragen von den überforderten Allgemeinmedizinern an den nächstbesten Psychiater überwiesen [51]. Der Sexualforscher Volkmar Sigusch (1940–2023) kritisierte dies nachdrücklich. Längst hatte sich gezeigt, dass das Hausarztsystem eigentlich nur in einer statischen Gesellschaft funktionieren konnte [52]. Der Zeitdruck in der Praxis verunmöglichte jedoch tiefergehende Anamnesen. Gleichwohl hielten die Funktionäre der Allgemeinmediziner an ihrer Vorstellung fest, ein bestandenes Staatsexamen genüge für die Ausübung der Praxis und Weiterbildungen seien im Grunde unnötig. Dies zeigte sich in besonders nachdrücklicher Weise, als der Hamburger Ordinarius für Psychiatrie Eberhard Schorsch (1935–1991) 1977 eine sexualmedizinische Ambulanz in das lokale Gesundheitssystem integrieren wollte [53]. In Hamburg hatte es seit Mitte der 1950er-Jahre Ärztefortbildungen in sexuellen Fragen gegeben, so dass der Kassenärztlichen Vereinigung theoretisch hätte bekannt sein müssen, wie groß der Bedarf an geschultem Personal war. Dies hinderte sie nicht daran, die Etablierung der Ambulanz nach Kräften zu verzögern. Ihre Einrichtung sei „unnötig“. Schorsch hatte eine Befragung von Hamburger Ärzten angestrengt, in deren Verlauf sich erwies, dass „sexuelle Therapie“ sich in der Verschreibung von Psychopharmaka und Hormonen erschöpfte [54]. Indirekt wies er dadurch nach, dass die Allgemeinmediziner damit ihr eigenes Ideal der 1920er-Jahre, nämlich eine Alternative zu den auf somatische Therapien festgelegten Fachärzten und den Versprechungen der pharmazeutischen Industrie darzustellen, längst hinter sich gelassen hatten. Die Verhältnisse hatten sich umgekehrt. Nicht mehr die Hausärzte waren die Vertrauenspersonen, sondern die Spezialisten. Daran konnte auch die Einführung der vierjährigen Weiterbildung zum „Allgemeinmediziner“ ab 1972 nichts mehr ändern [55].

## Die eigentlichen Folgen

Allerdings zeugte der Lösungsansatz von Paul Lüth und das Selbstbewusstsein der Gynäkologen von einem gewissen Sexismus. Die Übertragung der Sexualaufklärung von den Hausärzten auf die Frauenärzte implizierte die Gewissheit, dass nur Frauen, nicht aber Männer sexuelle Probleme hätten. Diese Überlegung harmonierte nicht mit der sozialen und gesellschaftlichen Wirklichkeit. Die sich entfaltende Frauenbewegung kritisierte zudem das Autoritätsverhältnis zwischen meist männlichen Frauenärzten und ihrer Patientenklientel. Davon konnten die Hausärzte nicht profitieren, die sich weiterhin in autoritativen Vorstellungen erschöpften. So wurde auf dem VII. Kongress der Akademie für Allgemeinmedizin 1976 in Graz festgehalten, der Allgemeinmediziner müsse die Frau in der Schwangerschaft „führen“, weil sie dazu selbst nicht in der Lage sei [56]. Darüber hinaus disqualifizierten sich die konservativen Fachvertreter, indem sie ihrem Kollegen Siegfried Ernst (1915–2001) in seinem Kampf gegen die Abtreibung folgten [57]. Ernst initiierte die „Europäische Ärzteaktion“ (EÄA) als radikales Vehikel zur Durchsetzung einer restaurativen Gesellschaftspolitik. Die sich mittlerweile generationenübergreifend durchsetzende sexuelle Revolution hatte zur Folge, dass sich zunächst im alternativen Sektor, dann aber auch in der Mitte der Gesellschaft ein Bewusstsein für Männergesundheit etablierte. Dies mündete zwanglos in der Stärkung der einzigen fachärztlichen Disziplin, die bislang sowohl von der Presse als auch den Funktionären der Ärzteverbände ignoriert worden war, den Urologen. Sie profitierten ab den frühen 1980er-Jahren von der Überheblichkeit der Allgemeinmediziner. Darüber hinaus nutzte den Urologen die Unfähigkeit der (heilpraktischen) Psychotherapeuten, bei Paartherapien somatische Probleme mit abzuklären. Der Anspruch der wenigen Sexualmediziner, beide Partner gleichermaßen zu behandeln, harmonierte nicht mit den gesellschaftlichen Anforderungen an den „deutschen Mann“, so dass Männer es vorzogen, einen für ihren Unterleib bestens ausgebildeten Spezialisten aufzusuchen. Eine nicht zu unterschätzende Rolle dürfte die Krankheit HIV/AIDS gespielt haben, die Relevanz von sexueller Gesundheit Männern zu verdeutlichen. Auch dies nutzte den Urologen, ihre Position innerhalb des Gesundheitssystems zu stärken. Nach der Einführung des Medikaments „Viagra“ 1998 kehrte das Thema Männergesundheit mit Wucht in die darauf kaum vorbereiteten Allgemeinpraxen zurück [58]. Längst haben sich die Hausärzte von der Idee verabschiedet, sie könnten oder sollten sexuell übertragbare Krankheiten behandeln [59]. Dies wird den Spezialisten überlassen.

In den nun zahlreicher erscheinenden Lehrbüchern der Urologie ab den 1970er-Jahren im deutschsprachigen Raum waren die Kapitel zu den „Erkrankungen der Genitalsphäre des Mannes“ [60] bzw. in Kapiteln zu den „Anomalien des unteren Harntraktes“, „Endokrinologie für Urologen“, die „Intersexualität“ oder „Fertilitätsstörungen“ sowie „Potenzstörungen und Climacterium virile“ unter dem Oberbegriff „Andrologie“ [61], auch „Venerologie für den Urologen“ [62], auf der Höhe der Zeit.

**Abb. 7:**
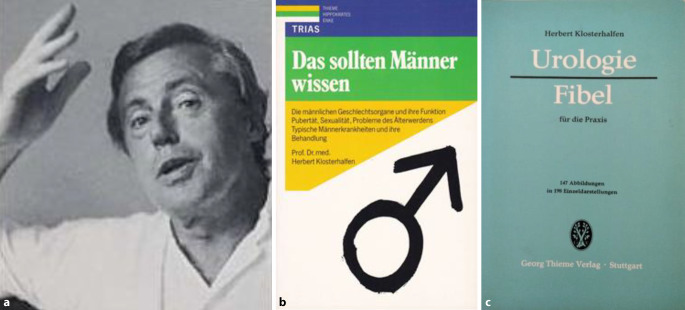
**a** Herbert Klosterhalfen (1925–2012), **b** Frontispiz Das sollten Männer Wissen, Thieme, Trias, **c** Urologie Fiebel, Thieme. (Repro Moll-Keyn, Sammlung Moll, mit freundl. Genehmigung)

In der Zeitschrift *Der Urologe A* und *B* (Springer; [63–65]), der Zeitschrift *Aktuelle Urologie* (Thieme, Stuttgart) oder auch der *Zeitschrift für Urologie* (VEB Georg Thieme, Leipzig; [66]) oder den Urologenkongressen [67] wie auch Einzelpublikationen im Taschenbuchformat [68, 69] waren die Themata ebenfalls entsprechend vertreten. Mehr denn je nutzte dies den Urologen, die somit zu den Profiteuren der sexuellen Revolution gehören und spätestens seit Beginn der 1990er-Jahre den gesamten Themenkomplex unter Einschluss hausärztlicher Aspekte unter der Überschrift „Männergesundheit“ abhandelten [70], auch wenn nicht alle Fachvertreter darüber immer begeistert waren. Daher rieten fortschrittliche Ordinarien der nächsten akademischen Urologengeneration dazu, in diesem Bereich besondere publizistische Anstrengungen zu unternehmen, jedoch diese Teildisziplin nicht ausschließlich zu bearbeiten, sondern mit weiteren Teilaspekten des Faches wie beispielsweise der Uroonkologie zu verbinden, da dieses Portfolio bei Überwiegen im Publikationsverzeichnis bei Lehrstuhlbesetzungen bei konservativen Fakultäten auch negativ bewertet werden könne. Daher wurde beispielsweise Themenkomplexe wie masturbatorisch eingeführte Blasenfremdkörper, die für das Fachgebiet seit jeher konstituierend waren, in Kapitel der Traumatologie „versenkt“ [71, 72]. Das war schon Ende der 1970er-Jahre so, als der Hamburger Ordinarius für Urologie Herbert Klosterhalfen (1925–2012) (Abb. 7) von der CDU als Experte rund um die Diskussion um ein Transsexuellengesetz angehört wurde [73].

Klosterhalfen wie auch der CDU war die gesamte sexualwissenschaftliche Forschung seit den 1920er-Jahren zu der Thematik restlos entgangen. Heute jedoch sind Urologen in die Transsexuellenbehandlung (vgl. AWMF-Leitlinie AWMF-Register-Nr. 138|001; [74]) fest eingebunden. Es bedarf also nicht zwingend der Kenntnis der eigenen Fachgeschichte, um von gesellschaftlichen Veränderungen zu profitieren.
